# Music with Different Tones Affects the Development of Brain Nerves in Mice in Early Life through BDNF and Its Downstream Pathways

**DOI:** 10.3390/ijms24098119

**Published:** 2023-05-01

**Authors:** Jing Wang, Jianxing Wang, Yulai Wang, Yiwen Chai, Haochen Li, Deyang Miao, Honggui Liu, Jianhong Li, Jun Bao

**Affiliations:** 1 College of Life Science, Northeast Agricultural University, Harbin 150030, China; 2 College of Animal Science and Technology, Northeast Agricultural University, Harbin 150030, China

**Keywords:** music, tone, neurodevelopment, BDNF-downstream pathways

## Abstract

As a means of environmental enrichment, music environment has positive and beneficial effects on biological neural development. Kunming white mice (61 days old) were randomly divided into the control group (group C), the group of D-tone (group D), the group of A-tone (group A) and the group of G-tone (group G). They were given different tonal music stimulation (group A) for 14 consecutive days (2 h/day) to study the effects of tonal music on the neural development of the hippocampus and prefrontal cortex of mice in early life and its molecular mechanisms. The results showed that the number of neurons in the hippocampus and prefrontal cortex of mice increased, with the cell morphology relatively intact. In addition, the number of dendritic spines and the number of dendritic spines per unit length were significantly higher than those in group C, and the expressions of synaptic plasticity proteins (SYP and PSD95) were also significantly elevated over those in group C. Compared with group C, the expression levels of BDNF, TRKB, CREB, PI3K, AKT, GS3Kβ, PLCγ1, PKC, DAG, ERK and MAPK genes and proteins in the hippocampus and prefrontal cortex of mice in the music groups were up-regulated, suggesting that different tones of music could regulate neural development through BDNF and its downstream pathways. The enrichment environment of D-tone music is the most suitable tone for promoting the development of brain nerves in early-life mice. Our study provides a basis for screening the optimal tone of neuroplasticity in early-life mice and for the treatment of neurobiology and neurodegenerative diseases.

## 1. Introduction

Enrichment environment is widely used in the fields of gene-environment interaction and plasticity of brain structure and function [1]. Previous studies have shown that abundant and suitable environments had positive impacts on the growth and development of animals [2]. Brosnan’s study shows that environmental enrichment induces white matter plasticity within the right frontoparietal network (and prevents age-related axonal dispersion) to promote the maintenance of neurocognitive health in later life [3]. Steele’s research results showed that music training during the periods of sensitive development could have a greater impact on brain structure and behavior than in other periods [4]. In the study of Wei, toys provided to lactating mice could increase the dendritic branches and excitatory synaptic connections of neurons in the CA3 region of the anterior limbic cortex and hippocampus [5]. Animals in the enrichment environments had high synaptic plasticity, which could help the body quickly cope with the negative effects of stress [6,7,8]. In conclusion, the enrichment environment in the critical period when the nervous system is prone to change can regulate the brain’s physiological environment and promote the development of brain neural networks and brain function.

Nowadays, music enrichment environment is regarded as a suitable environment in the study of emotional and neuroplasticity [9,10]. Music influences brain structural changes in specific musical ways [11]. Previous studies have shown that rodents exposed to music tend to have enhanced neurogenesis and neuroplasticity [12]. Male SD rats exposed to Mozart K.448 increased hippocampal spinal nerve density at 21–76 days postpartum (12h/d) [13]. Maguire pointed that Mozart’s music (K.448) might alert protective brain networks to produce higher brain frequencies, thus exerting an inhibitory effect on seizures via GABA interneurons [14]. Kim’s study also showed that music promoted the neural development of the hippocampus in rats [15]. Prenatal music enrichment environment promoted motor and somatosensory cortical neural development and increased cortical thickness in young rats [16]. Music has many benefits in the development of the cerebral nervous system [17], but whether different tones are the key factor in the influence of musical effects on the neural development of animals is unclear.

Neurons are the basic units of the brain and nervous system [18]. Dendrites are branchlike structures emanating from the cell bodies of neurons. Dendrites contain multiple receptors that could accept nerve impulses from other neurons. A study has shown that dendritic spines were the transmission sites of excitatory synapses, which could receive information and form synaptic connections, and were considered the basis of synaptic plasticity [19]. Studies have shown that dendritic spines and synapses could sensitively reflect the impact of the living environment on organisms [20,21,22]. Environmental enrichment caused an increase in dendritic branching in Wistar rats and an increase in dendritic spine density in layer-III parietal pyramidal neurons [23]. Aghighi also noted that environmental enrichment restores synaptic plasticity in prenatally stressed rats [24]. Stein’s study shows that shorter environmental enrichment could also alter synaptic plasticity and molecular markers related to cognitive function in older animals [25]. Reduced brain-derived neurotrophic factor (BDNF) expression levels and abnormalities in BDNF-related pathways could induce neuropathic diseases (literature added) [26,27]. The combination of BDNF with its tyrosine kinase receptor B (TRKB) could mediate mitogen-activated protein kinase (MAPK)/extracellular response kinase (ERK), phosphoInositide-3 kinase (PI3K)/protein kinase B (AKT) and phospho-lipase C (PLCγ1)/protein kinase C (PKC) signaling pathways. All of these pathways could regulate cell proliferation and differentiation and thus affect the development of neurons. In addition, the cAMP-response element binding protein (CREB) is a key regulatory factor downstream of the ERK signaling pathway. CREB could directly regulate the expression of BDNF after being activated. But whether different tones regulate neurodevelopment through BDNF and its downstream pathways is unclear.

Tone was introduced by the American National Standards Institute (ANSI) and was defined as “an auditory perceptual property induced by the sound that could be arranged from low to high”. Different frequencies are key factors in determining different tones, and different musical pitches have different emotional tones. The ancient Greeks considered the tone of A-tone to be high, G-tone to be buoyant and D-tone to be warm. Music as a regular sound could enrich the auditory environment of domesticated animals and has many benefits in terms of brain-nervous system development [17]. However, there are few articles in research that use tone as a variable factor in the study, and the mechanisms of the effect of musical structure on the organism have not been studied in depth. The present experiment was conducted to investigate whether the tone is an important musical element in producing musical effects, using tone as the basis of the study.

In this study, mice were selected as the research object, and the effects of different tone music enrichment environments on the hippocampus and prefrontal cortex, dendritic spines, synaptic connections and BDNF/TRKB/CREB, MAPK/ERK, PI3K/AKT and PLCγ1/PKC signaling pathways were explored to examine the molecular mechanism of the influence of musical tone characteristics on the neurodevelopment of mice so as to provide a reference for screening the optimal tone affecting the neuroplasticity in the early-life of mice and to provide a theoretical basis for the treatment of neurodegenerative diseases.

## 2. Results

### 2.1. Effects of Music in Different Tones on Nissl Bodies in the Hippocampus and Prefrontal Cortex

**Figure 1:**
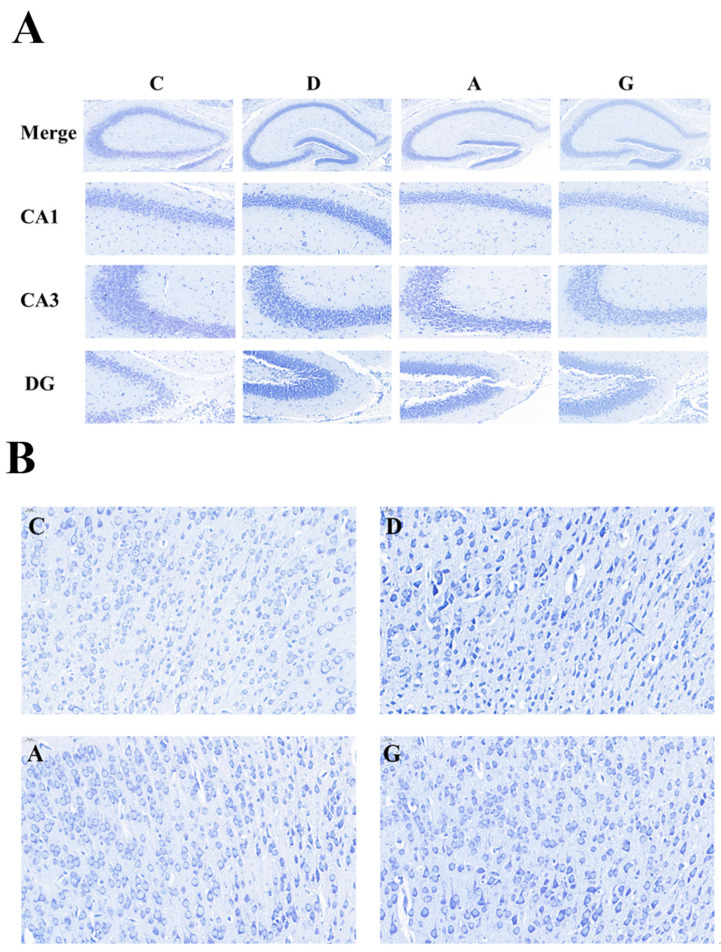
Effects of music in different tones on Nissl Staining. (**A**) In hippocampus. (**B**) In prefrontal cortex.

Compared with group C, in the hippocampus (Figure 1A) and prefrontal cortex (Figure 1B) in the music groups, pyramidal cells were arranged in an orderly way with relatively complete cell morphological shrinkage. The nuclear pyknosis phenomenon was less, and there was no shallow coloring and partial Nissl dissolution or disappearance. The cytoplasm was blue, and the Nissl staining was deeper.

### 2.2. Effects of Music in Different Tones on Neuronal Dendritic Development in the Hippocampus and Prefrontal Cortex

**Figure 2:**
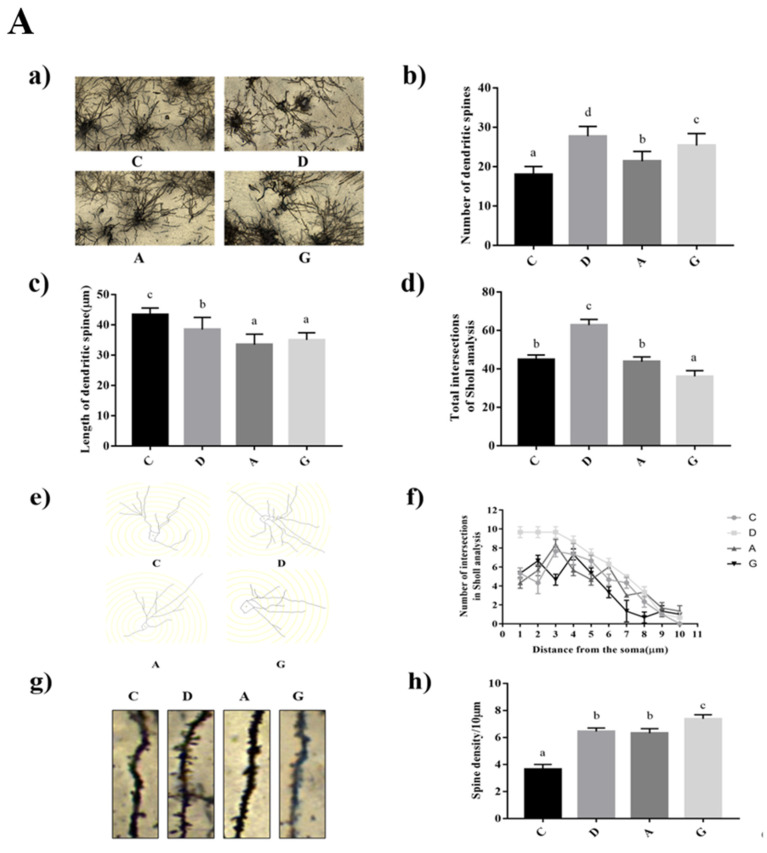

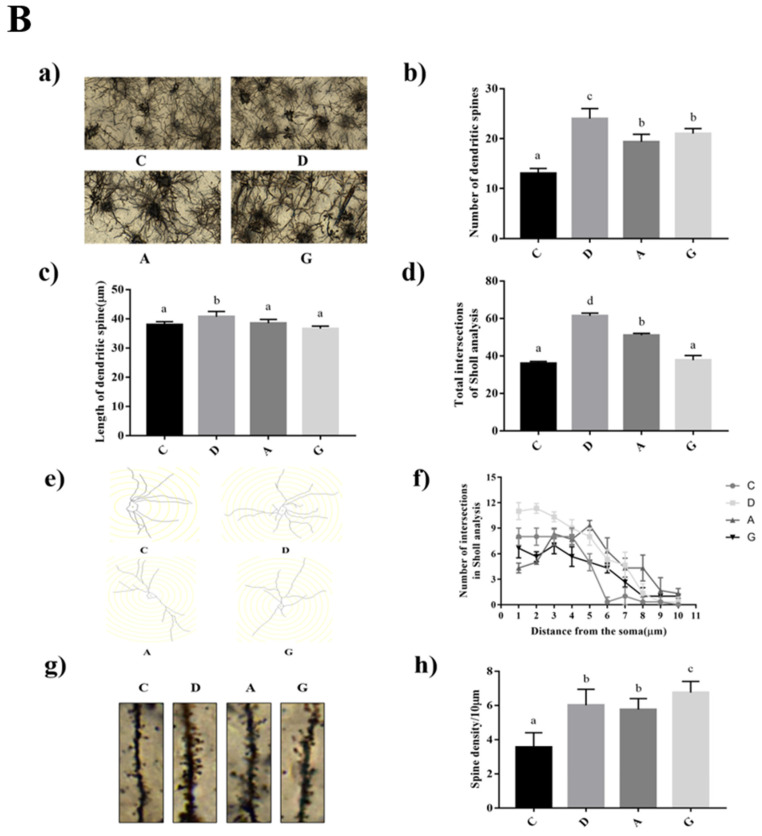
Effects of music in different tones on the development of neuronal dendrites. (**A**). In hippocampus. (**B**). In prefrontal cortex. Data in each group were described as Mean ± SD. (a) Results of Golgi staining in hippocampus of each group, (b) Number of dendritic spines of each group, (c) Length of dendritic spine of each group(µm), (d) The number of intersection points between dendritic spines and concentric circles in each group, (e) Total intersections of shall analysis of each group, (f) Number of intersections in shall analysis, (g,h) Dendritic spine density within 10 microns in each group. (a–d) shows that bars with different lowercase represent statistical significance between two groups (*p* < 0.05).

In the hippocampus, the number of dendritic spines and the number of dendritic spines per unit length in the music groups were significantly higher than those in group C (*p* < 0.05). The length of hippocampal dendritic spines in the music groups was significantly lower than that in group C (*p* < 0.05) (Figure 2A). The number of intersection points between dendrites and concentric circles in group D was significantly higher than that in other groups (*p* < 0.05), but there was no significant difference in the number of intersection points between group G, group A and group C (*p* > 0.05) (Table S2).

In the prefrontal cortex, the number of dendritic spines in the music groups was significantly higher than that in group C (*p* < 0.05), and the length of dendritic spines in group D was significantly higher than that in the other groups (*p* < 0.05). The number of dendritic spines per unit length in group G was significantly higher than that in other groups (*p* < 0.05) (Figure 2B). The number of intersection points between dendrites and concentric circles in group D and group G was significantly higher than that in group C and group A (*p* < 0.05), but there was no significant difference in the number of intersection points between group A and group C (*p* > 0.05) (Table S3).

### 2.3. Effects of Music in Different Tones on the Expression of Synaptic Protein in the Hippocampus and Prefrontal Cortex

**Figure 3:**
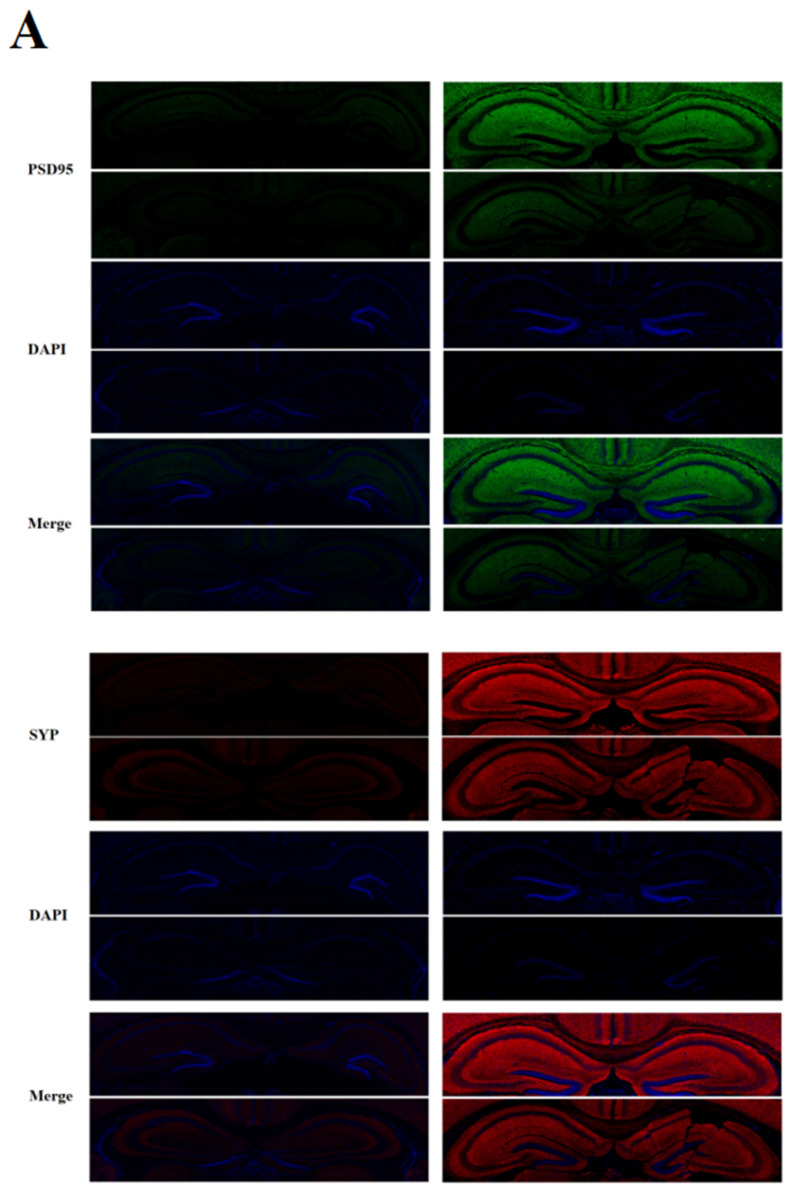

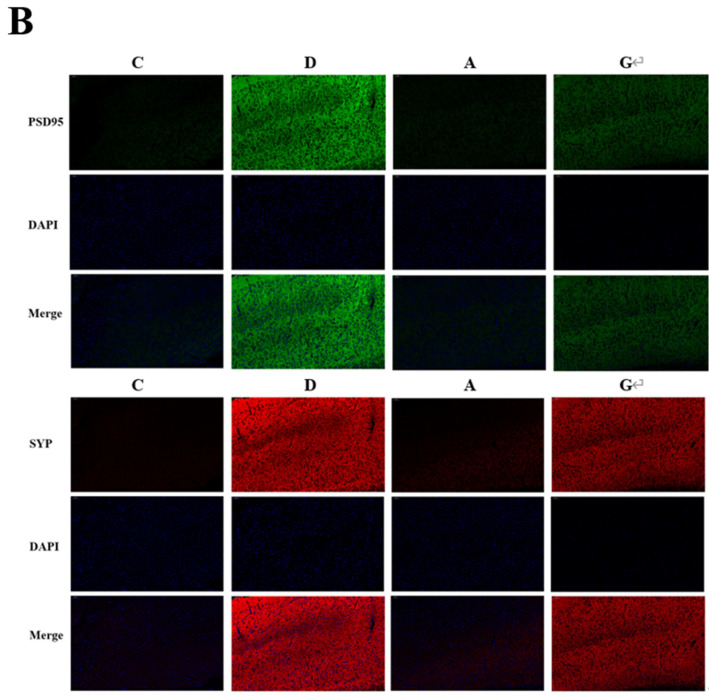
Effects of music in different tones on synaptic protein expression (200 μm). (**A**). In hippocampus. (**B**). In prefrontal cortex. Data in each group were described as Mean ± SD. Bars with different lowercase represent statistical significance (*p* < 0.05) between two groups.

In the hippocampus (Figure 3A) and prefrontal cortex (Figure 3B) of mice, the protein expression levels of PSD95 and SYP in the music groups were significantly higher than those in group C (*p* < 0.05). The protein expression levels of PSD95 and SYP in group A and group G were significantly lower than those in group D (*p* < 0.05). The fluorescence intensity of PSD95 and SYP in group G was significantly higher than that in group A (*p* < 0.05). The positive rates of PSD95 and SYP were shown in Table S3.

### 2.4. Effects of Music in Different Tones on mRNA and Protein Expression Levels of BDNF/TRKB/CREB Pathways in the Hippocampus and Prefrontal Cortex

**Figure 4:**
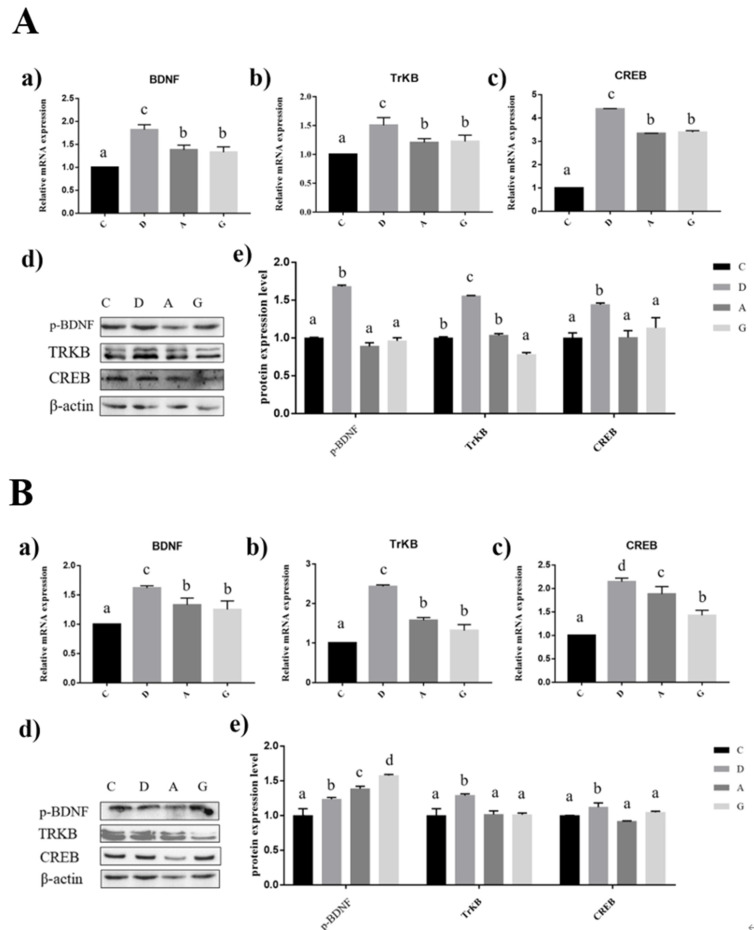
Effects of music in different tones on mRNA and protein expression levels of the BDNF/TRKB/CREB pathway. (**A**). In hippocampus. (**B**). In prefrontal cortex. (a–c) showed that the bar diagram of BDNF, TrKB and CREB genes. (d,e) showed that the bar diagram of BDNF, TrKB and CREB proteins. The different lowercase letters at the top of the bar chart indicated significant statistical differences among different groups (*p* < 0.05).

In the hippocampus of mice, the expression levels of BDNF, TRKB and CREB mRNA in music groups were significantly higher than those in group C (*p* < 0.05), and the expression levels of BDNF, TRKB and CREB mRNA in group D were significantly higher than those in group A and group G (*p* < 0.05); however, there was no significant difference between group A and group G (*p* > 0.05). The expression levels of BDNF, TRKB and CREB protein in group D were significantly higher than those in the other three groups (*p* < 0.05), but there were no significant differences in the expression levels of BDNF and CREB protein in the other three groups (*p* > 0.05). The expression level of TRKB protein in group G was significantly lower than that in other groups (*p* < 0.05), but there was no significant difference between group A and group C (*p* > 0.05) (Figure 4A).

In the prefrontal cortex of mice, the expression levels of BDNF, TRKB and CREB mRNA in the music groups were significantly higher than those in group C (*p* < 0.05). The expression levels of BDNF, TRKB and CREB mRNA in group D were significantly higher than those in the other three groups (*p* < 0.05). The expression levels of BDNF and TRKB mRNA were not significantly different between group A and group G (*p* > 0.05). The expression level of CREB mRNA in group A was significantly higher than that in group G (*p* < 0.05). The expression level of BDNF protein in group C was significantly lower than that in the music groups (*p* < 0.05). The protein expression level of BDNF in group G was significantly higher than that in the other three groups (*p* < 0.05), and that in group A was significantly higher than that in group D. The expression levels of TRKB and CREB protein in group D were significantly higher than those in the other three groups (*p* > 0.05), but there was no significant difference among the other three groups (*p* > 0.05) (Figure 4B).

### 2.5. Effects of Music in Different Tones on mRNA and Protein Expression Levels of PLCγ1/PKC Pathways in the Hippocampus and Frontal Cortex

**Figure 5:**
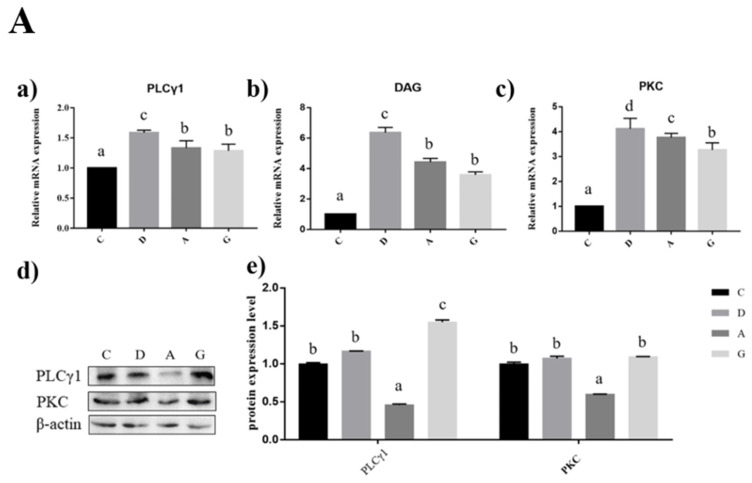

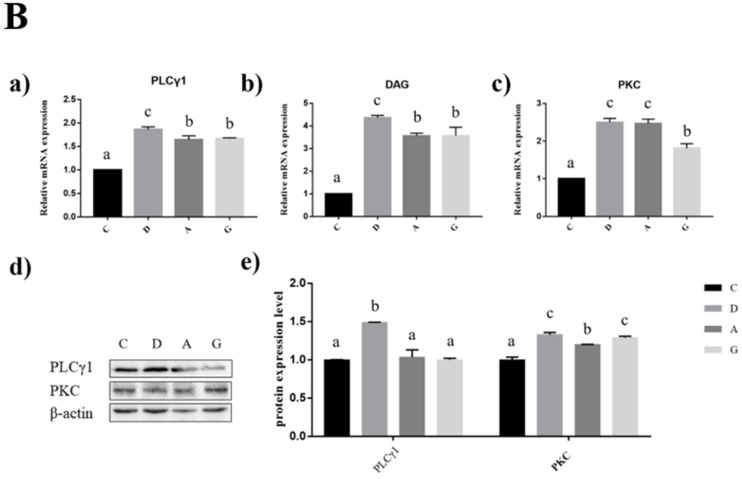
Effects of music in different tones on mRNA and protein expression levels of the PLCγ1/PKC pathway. (**A**). In hippocampus. (**B**). In prefrontal cortex. (a–c) showed that the bar diagram of PLCγ1, DAG and PKC genes. (d,e) showed that the bar diagram of PLCγ1 and PKC proteins. The different lowercase letters at the top of the bar chart indicated significant statistical differences among different groups (*p* < 0.05).

In the hippocampus of mice, the expression levels of PLCγ1, DAG and PKC mRNA in the music groups were significantly higher than those in group C (*p* < 0.05). The PLCγ1, DAG and PKC mRNA expression levels in group D were significantly higher than those in the other three groups (*p* < 0.05). The expression levels of PLCγ1 and DAG mRNA were not significantly different between group A and group G (*p* > 0.05). The expression level of PKC mRNA in group A was significantly higher than that in group G (*p* < 0.05). PLCγ1 protein expression level in group G was significantly higher than that in the other three groups (*p* < 0.05); PLCγ1 protein expression level in group A was significantly lower than that in the other three groups (*p* < 0.05); and there was no significant difference between group D and group C (*p* > 0.05). The protein expression level of PKC in group A was significantly lower than that in the other groups (*p* < 0.05), but there was no significant difference among the other three groups (*p* > 0.05) (Figure 5A).

In the prefrontal cortex of mice, the expression levels of PLCγ1, DAG and PKC mRNA in the music groups were significantly higher than those in group C (*p* < 0.05). The PLCγ1, DAG and PKC mRNA expression levels in group D were significantly higher than those in the other three groups (*p* < 0.05). The PLCγ1 protein expression level in group D was significantly higher than that in the other three groups (*p* < 0.05), but there was no significant difference among the other three groups (*p* > 0.05). The PKC protein expression level of group C was significantly lower than that of the other three groups (*p* < 0.05); the PKC protein expression level of group A was significantly lower than that of group D and group G (*p* < 0.05); and there was no significant difference between group D and group G (*p* > 0.05) (Figure 5B).

### 2.6. Effects of Music in Different Tones on the mRNA and Protein Expression Levels of PI3K/AKT Pathways in the Hippocampus and Prefrontal Cortex

**Figure 6:**
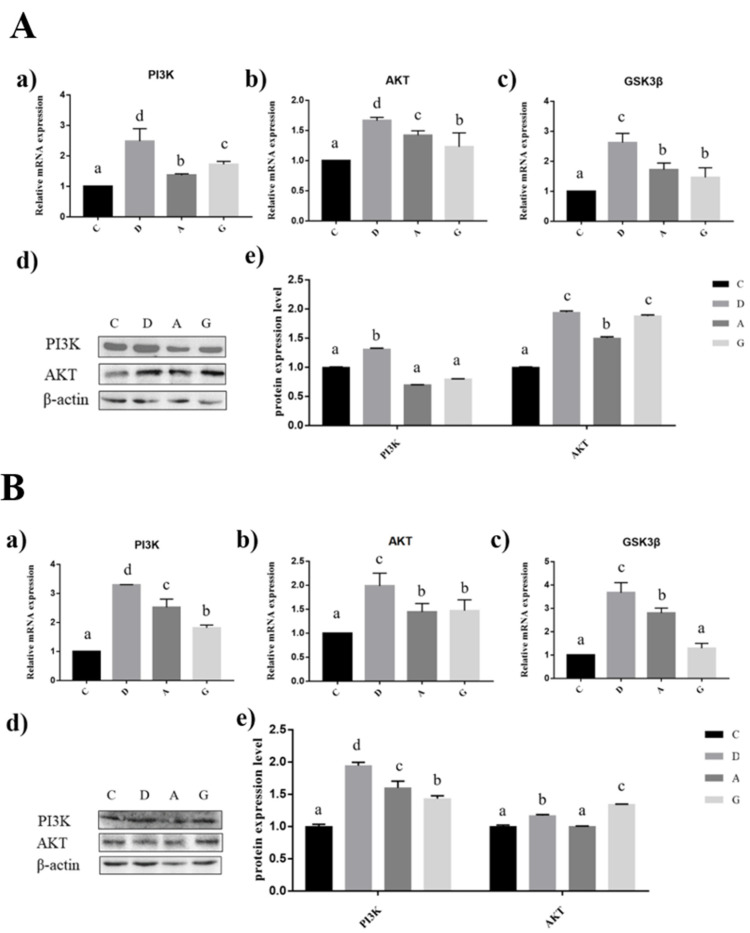
Effects of music in different tones on mRNA and protein expression levels of the PI3K/AKT pathway. (**A**). In hippocampus. (**B**). In prefrontal cortex. (a–c) showed that the bar diagram of PI3K, AKT and GS3Kβ genes. (d,e) showed that the bar diagram of PI3K and AKT proteins. The different lowercase letters at the top of the bar chart indicated significant statistically differences among different groups (*p* < 0.05).

In the hippocampus of mice, the expression levels of PI3K, AKT and GS3Kβ mRNA in the music groups were significantly higher than those in group C (*p* < 0.05), and the expression levels of PI3K, AKT and GS3Kβ mRNA in group D were significantly higher than those in group A and group G (*p* < 0.05). The PI3K protein expression level in group D was significantly higher than that in the other three groups (*p* < 0.05), but no significant difference among the other three groups was observed (*p* > 0.05). The expression level of AKT in the music-treated groups was significantly higher than that in group C (*p* < 0.05), and the expression level of AKT in group A was significantly lower than that in group D and group G (*p* < 0.05); however, there was no significant difference between group D and group G (*p* > 0.05) (Figure 6A).

In the prefrontal cortex of mice, the mRNA expression levels of PI3K, AKT and GS3Kβ in group D were significantly higher than those in the other three groups (*p* < 0.05); the PI3K and AKT in group C were significantly lower than those in group A and group G (*p* < 0.05); and the PI3K mRNA expression in group A was significantly higher than that in group G (*p* < 0.05). The mRNA expression level of AKT was not significantly different between group A and group G (*p* > 0.05). The GS3Kβ mRNA expression level of group C and group G was significantly lower than that of group A (*p* < 0.05), and there was no significant difference between group C and group G (*p* > 0.05). The PI3K protein expression level of group D was significantly higher than that of the other three groups (*p* < 0.05); the PI3K protein expression level of group A was significantly lower than that of the other three groups (*p* < 0.05); and group A was significantly higher than group G (*p* < 0.05). The expression level of AKT in group G was significantly higher than that in the other three groups (*p* < 0.05), and that in group D was significantly higher than that in group C and group A (*p* < 0.05); however, there was no significant difference between group C and group A (*p* > 0.05) (Figure 6B).

### 2.7. Effects of Music in Different Tones on the mRNA and Protein Expression Levels of MAPK/ERK Pathway in the Hippocampus and Frontal Cortex

**Figure 7:**
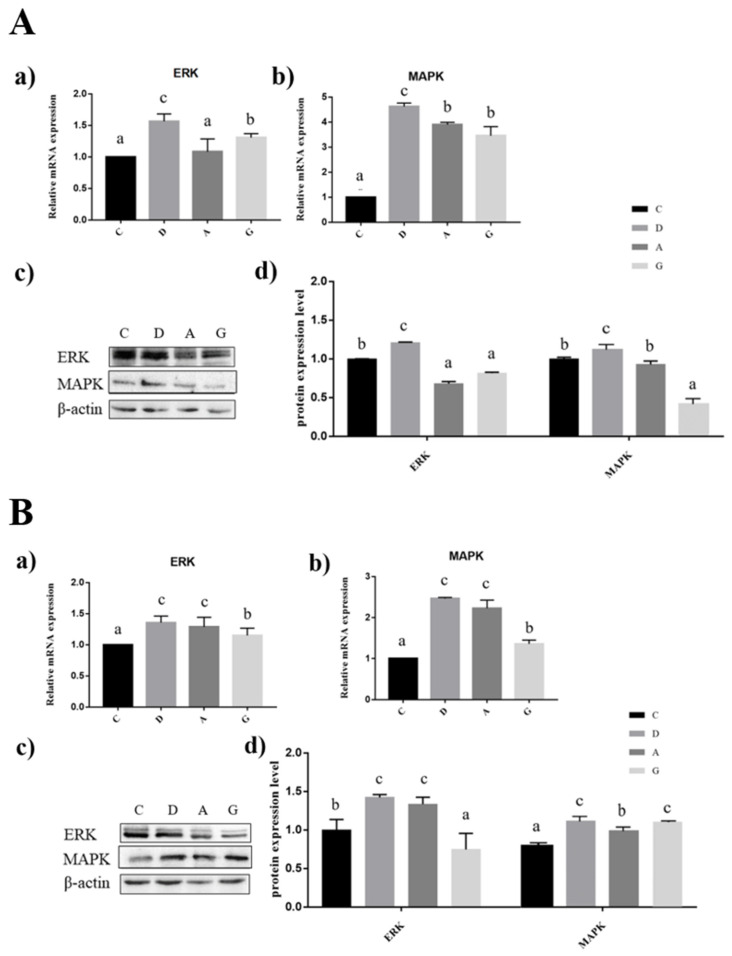
Effects of music in different tones on mRNA and protein expression levels of the MAPK/ERK pathway. (**A**). In hippocampus. (**B**). In prefrontal cortex. (a,b) showed that the bar diagram of ERK and MAPK genes. (c,d) showed that the bar diagram of ERK and MAPK proteins. The different lowercase letters at the top of the bar chart indicated significantly statistical differences among different groups (*p* < 0.05).

In the hippocampus of mice, the expression levels of MAPK and ERK mRNA and proteins in group D were significantly higher than those in the other three groups (*p* < 0.05). The expression level of ERK mRNA in group G was significantly higher than that in group C and group A (*p* < 0.05), but there was no significant difference between group C and group A (*p* < 0.05). The expression level of MAPK mRNA in group C was significantly lower than that in group G and group A (*p* < 0.05), but there was no significant difference between group G and group A (*p* < 0.05). The expression level of ERK protein in group C was significantly higher than that in group G and group A (*p* < 0.05), but there was no significant difference between group G and group A (*p* < 0.05). The expression level of MAPK mRNA in group G was significantly lower than that in group C and group A (*p* < 0.05), but there was no significant difference between group C and group A (*p* < 0.05) (Figure 7A).

In the prefrontal cortex of mice, the expression levels of MAPK and ERK mRNA in group A were significantly lower than those in the other three groups (*p* < 0.05). The expression levels of MAPK and ERK mRNA in group G were significantly lower than those in group D and group A (*p* < 0.05), but there was no significant difference between group D and group A (*p* < 0.05). The expression level of ERK protein in group D and group A were significantly higher than that in other groups, and the expression level of ERK protein in group C was significantly higher than that in group G (*p* < 0.05); however, there was no significant difference between group D and group A (*p* < 0.05). The expression level of MAPK protein in group D and group G were significantly higher than that in other groups (*p* < 0.05), the expression level of MAPK protein in group C was significantly higher than that in group A (*p* < 0.05), but there was no significant difference between group D and group G (*p* < 0.05) (Figure 7B).

## 3. Discussion

Survival experience and environment could affect neural development [28]. Tone is the most important perceptual feature for the auditory system to perceive sound stimulus, and its determined factors depend on the frequency of sound. Studies have shown that tone could cause differences in musical perception in organisms [29]. Enriched and suitable music environments promote neural development [28]. These results of our study showed that the length of dendrites and the number of dendritic spines increased in the hippocampus and prefrontal cortex of mice exposed to different musical tones for 2 h per day. Previous studies showed that enrichment environment could increase the number of synapses and dendritic spines in hippocampal neuronal units in rats, promote hippocampal neurogenesis, reduce cell apoptosis, and glial cell regeneration and synaptogenesis significantly increase [30,31,32]. The results of previous reports were similar to those of this experiment. In this study, the increase of neuronal dendrites and dendritic spines in the music environment means that neurons in the hippocampus and prefrontal cortex could project to more areas, and more synaptic connections could be established between neurons [19], which is conducive to improving the information transmission of the nervous system. SYP and PSD95 are important proteins involved in the regulation of synaptic plasticity [33]. The present study showed that music environment increased the expression of SYP and PSD95 proteins. Kozorovitskiy’s study showed that the levels of SYP and PSD95 proteins in hippocampus, forebrain, hypothalamus and prefrontal cortex were up-regulated under the influence of enrichment environment [20]. It was speculated that the increase of dendritic spines in the music groups was closely related to the increase of SYP and PSD95 proteins in the prefrontal cortex and hippocampus. The effects of the D-tone enrichment environment on the hippocampus and prefrontal cortex were most obvious, suggesting that the D-tone environment might be the most suitable tone for promoting neural development in mice in this study.

Except for the changes in neurons, the effects of music on the signal transduction of BDNF and its downstream pathways were studied. The results showed that the expression level of BDNF increased significantly in the music groups. Acoustic stimulation increased BDNF expression in the hippocampus of wild-type adult mice and perinatal chicks [34,35]. Chikahisa’s study showed that perinatal exposure to music modulated the BDNF/TRKB pathway in mice [36]. p-BDNF is a phosphorylated protein of BDNF and a prerequisite for the function of BDNF. Taherian’s study suggested that the expression levels of p-BDNF protein increased could promote activation of the hippocampal in rats [37]. The results of this study were consistent with the results of previous studies, and it is speculated that the improvement of the changes in neural structure and function in an enrichment music environment is based on the changes of BDNF. Studies have shown that BDNF could activate TRKB receptors, and the BDNF/TRKB pathway plays a fundamental role in mediating lasting changes in the function of central synapses [38,39,40,41] and in the dendrite structure of neurons [42,43,44]. In the study reported by Zheng, the number of PV neurons in the hippocampal CA1 region of mice aged 4 weeks and 6 weeks after TRKB knockout was reduced [45]. In this study, TRKB expression was significantly elevated in the music-treated groups, suggesting that the TRKB is one key factor in the changes of neural structure and function in the enrichment music environment. This study showed that the increased expression levels of BDNF and TRKB had the same trend as the morphological changes of neurons in the hippocampus and prefrontal cortex, suggesting that music could regulate the neural development of the hippocampus and prefrontal cortex through the BDNF/TRKB axis.

BDNF binding to TRKB is followed by receptor dimerization and kinase activation. Subsequently, phosphorylated cytoplasmic tyrosine provides docking sites for the initiation of two intracellular signaling pathway bridging proteins, including PLCγ1/PKC [46], MAPK/ERK and PI3K/AKT, which are major effectors of neurotrophic factor [47,48,49]. It could promote nerve cell survival and process growth [50]. The results of this study showed that different tones of music increased the expression of PLCγ1, DAG and PKC mRNA in the hippocampus and prefrontal cortex in the early-life of mice, and the protein levels of PLCγ1 and PKC were significantly increased. It was speculated that the increased expression levels of PLCγ1 and PKC were closely related to the increase of dendrites and dendritic spines of neurons in the hippocampus and prefrontal cortex. Music could promote the survival and growth of mouse neurons through the PLCγ1/PKC axis. The PI3K/AKT signaling pathway as the downstream signaling pathway of BDNF/TRKB has attracted extensive attention from scholars [51,52,53]. Reduction of PI3K could inhibit the long-term enhancement of dentate gyrus in the hippocampus of rats [54]. The results of this study showed that different tones of music increased the expression of PI3K and AKT mRNA and proteins in the hippocampus and prefrontal cortex in the early-life of mice, which is consistent with the study of Li. Li et al. revealed that, when the expression of BDNF increased, the biological activity of the PI3K/AKT signaling pathway was also enhanced, resulting in significant improvement of sensory and motor functions in sciatic nerve transection mice [55]. It is speculated that music environment might maintain nerve growth and development and protect damaged neurons by activating the PI3K/AKT axis. The MAPK/ERK pathway is also the downstream pathway of BDNF/TRKB [46], which plays an important role in physiological processes such as proliferation, differentiation and synaptic plasticity of nerve cells [56]. Carlini found that fluoxetine could down-regulate the cognitive function of mice through MAPK/ERK signaling pathway [57] and increase the number of new neurons after ischemia injury in the dentate gyrus of the hippocampus [58]. The results of this study showed that the levels of MAPK and ERK mRNA in the music groups increased to varying degrees, which was consistent with previous studies. Activated ERK can translocate from the cytoplasm to the nucleus, activating RSK2, which then phosphorylates the transcription factor cAMP response element binding protein (CREB) on serine. Phosphorylated CREB binds to the cAMP response element (CRE) DNA promoter site to initiate gene transcription [59]. The results of this study showed that CREB was significantly increased. It is speculated that different tones of music could regulate neuronal cell survival, proliferation and differentiation through MAPK/ERK/CREB pathway and promote brain nerve development. This is the first study to investigate the morphological and molecular biological mechanisms of the beneficial effects of different tones on neuronal plasticity.

The music environment provided new stimuli to mice, which could pass into the brain through the sensory system. The neurons in the brain would undergo biochemical reactions, and the connections between the brains would also increase. The original two independent neurons might activate each other due to the new stimuli, and some connections would be established between the neurons. Learning new information could increase the number of dendrites. The stimulation continues, and the connection between the two neurons stabilizes, forming a memory and creating a learning effect. In this study, we found that the music enrichment environment led to an increase in neuronal branching, density and synapses in the hippocampus and prefrontal cortex, in concert with an increase in BDNF and its downstream pathways related factors (Figure S1). It is worth noting that the D-tone music environment had the most significant effect on BDNF and its downstream pathways-related factors in the hippocampus and prefrontal cortex in the early-life of mice, indicating that the D-tone music environment had a more positive regulatory effect on BDNF and its downstream pathways. In this study, life events such as feeding in a normal environment and a music-enriched environment affected the density, length and number of dendritic spines of neurons in the hippocampus and prefrontal cortex, the changes of neuronal morphology and gene expression levels of related pathways. This study suggests that the developmental system of neurons is environmentally dependent. However, the effects of long-term musical stimulation on the neural development and learning level of mice are still unclear, which is worthy of further study.

## 4. Materials and Methods

### 4.1. Feeding and Management of Experimental Animals

Mice were purchased from Liaoning Changsheng biotechnology Co., Ltd. (Benxi, China). Mice were maintained on a 12-hour-light/12-hour-dark cycle (lights on at 08:00) at a controlled ambient temperature (23 ± 1 °C) and humidity (50%–60%). All procedures used in the experiment were in line with the guidance on protecting animals used for scientific purposes (2010/63/EU) and were approved by the Institutional Animal Care and Use Committee of Northeast Agricultural University (No. SRM-06). During the test, the mice could drink water and eat freely (Table S1). The wood bedding was changed every four days. Animal feeding and immunization procedures were carried out in accordance with the breeding management rules.

### 4.2. Grouping for Experimental Animals

Mice were randomly assigned to four groups: silence, which did not accept any music stimulation (control group); D-tone of Mozart’s piano sonata, K.448, which is usually used in studies on the “Mozart’s effect” (Group D); A-tone of Mozart’s piano sonata, K.448, (Group A); G-tone of Mozart’s piano sonata, K.448, (Group G). Music was played only throughout the dark period (20:00 to 22:00 every day), but not the light period, so that the mice would not be disturbed in their sleep. At the same time, each processing unit was equipped with audio equipment with left and right channels, and the sound level of music was kept at 65dB in home cages. During the music playing, the position of the radio recorder and mice and the intensity of the music remained constant.

### 4.3. Sample Collection

One hundred and twenty mice (14 days old) were sacrificed to measure each gene and protein level. The entire brain was excised and dissected into two brain regions (consisting mainly of the prefrontal cortex and hippocampus). Samples were immediately frozen on dry ice and stored at −80 °C refrigerator. At the time of decapitation, the whole brains were collected in a test tube, which was filled with 4% paraformaldehyde for morphological examination.

### 4.4. Nissl Staining

Brain tissues were immersed in 4% paraformaldehyde. Brain tissues were removed from 4% paraformaldehyde and immersed in 20% sucrose and 30% sucrose solutions successively. After completing the dehydration process the entire brain was serially coronal sectioned (25 µm) using a frozen sectioning machine. The brain sections were attached to gelatin film and left at room temperature for 24 h. After dehydration and rehydration operations, the sections were stained with 0.5% cresyl violet (Solarbio, Shanghai, China) (20 min); immersed in distilled water (30 s), 95% ethanol (2 min), 100% ethanol (2 min) and xylene (10 min) in sequence; and, finally, sealed with neutral resin. Panoramic images of brain tissue were obtained by panoramic multilayer scanning using a light microscope (BX53, Olympus, Tokyo, Japan).

### 4.5. Golgi Staining

Brain tissues were removed from 4% paraformaldehyde, submerged in Golgi staining solution, soaked for 48 h and then replaced with new staining solution every 2 days for a total of 14 days. The whole brains were removed and immersed in distilled water (3 times), 80% glacial acetic acid, distilled water and 30% sucrose solution, respectively. Subsequently, frozen serial coronal sections (100 μm) were made, and the brain slices were attached to gelatin slides and protected from light overnight. Brain slices were treated with 12% ammonia, staining solution fixative, gradient ethanol and xylene, respectively, and then sealed with neutral gum. Finally, photographs were taken using a light microscope and a camera. Sholl analysis was performed using Image J software, and the number of dendritic branches was evaluated by the total number of intersections and the distribution of Sholl intersections.

### 4.6. Immunofluorescence

The brain sections were washed three times with 0.01M PBS solution; then the sections were placed in 4% polyformaldehyde for 20 min. After that, they were sealed with 3% hydrogen peroxide for 20 min. Finally, the first step was repeated three times. Completing the above steps, the brain tablet was incubated for 1 h in a closed buffer containing 10% normal goat serum and 0.1% TritionX-100. The closure was completed; the PSD95 (1:1000, Cell Signaling Technolo Gy, Inc., Boston, USA) and SYP (1:800, Cell Signaling Technology, Inc., Boston, USA) antibodies spent the night in the 4 °C environment. The next day, the purpose of the 0.01M Phosphate Buffered Saline (PBS) solution was cleaned three times to remove uncombined anti-distance. After staining, the anti-quenching agent droplets were added to the slice and then sealed. Panoramic images of brain tissue were obtained using a digital slicing scanner panoramic multilayer. Finally, Image J software was used to measure the integral light density and fluorescence colocation analysis.

### 4.7. Real-Time Quantitative PCR (qRT-PCR)

The TRIzol (Invitrogen, Carlsbad, CA, USA) method was used to extract the total RNA in the hippocampus and the prefrontal cortex. RNA concentration and purity were determined by micro-spectrophotometer (Hangzhou All Sheng Instruments Co., Ltd., Hangzhou, China). Then the instructions of the M5 Sprint qPCR RT kit (with gDNA remover) (Mei5 Biotechnology Co. Ltd., Beijing, China) were used to synthesize cDNA. β-actin was used as an internal reference gene. LightCycler480 System real-time quantitative PCR instrument (Roche Diagnostics, Mannheim, Germany) was used for the fluorescence signal acquisition. qRT-PCR was carried out in a 10 μL reaction system, including FastStart Universal SYBR Green Master (ROX) 3.4 μL, forward primer 0.3 μL, reverse primer 0.3 μL (Shanghai Sangon Biotech, Shanghai, China), cDNA 1 μL, DEPC water supplement to 10 μL [60,61]. The calculation formula was as follows:

Relative expression of mRNA = 2^−[(CT treatment group-CT treatment group reference)−(CT control group-CT control group reference)]^.

### 4.8. Western Blot

A 100mg tissue sample was ground in a refrigerated homogenizer and mixed with 1mL cell lysis solution (Beyotime, Beijing, China), which contained proteases and phosphatase inhibitors (Beyotime, China). According to the kit instruction manual, the sample concentration was measured by using bicinchoninic acid (BCA) protein concentration (Beyotime, China). Total protein was concentrated in sodium dodecyl sulfate-polyacrylamide gel electrophoresis at 100V for 30 min and then separated in the 10% separation glue for 90 min. Marker and total protein were transferred to polyvinylidene fluoride (PVDF) membrane (Cytiva, Marlborough, MA, USA) at 200 mA for 90 min. PVDF membrane was blocked in 5% skimmed milk at 37 °C for 2 h. PVDF membrane was placed in the corresponding diluted primary antibody and incubated at 4 °C for 12 h. Then the PVDF membrane was placed in the horseradish peroxidase-labeled goat antirabbit IgG and incubated at room temperature. After using enhanced chemiluminescence (ECL) chromogenic solution (Beyotime, Beijing, China) to treat the strip, chemical imaging system (Syngene G: box chemi XX9, UK) was used to detect the signal bands. Image J 18.0 software (National Institutes of Health, Bethesda, MD, USA) was used to measure the relative gray values [62,63]. The calculation formula was as follows:

Relative expression of protein = (processing group target protein ÷ treatment group internal parameter protein) ÷ (comb control group target protein ÷ control group internal reference protein)

### 4.9. Statistical Analysis

Nissl staining, Golgi staining and immunofluorescence were statistically analyzed by GraphPad Prism software. Data were expressed as Mean ± SD. Shapiro-Wilk was used to perform normality tests before one-way ANOVA, and the total number of Sholl intersections, dendrite spine density and SYP and PSD95 expression were compared by one-way ANOVA. *p* value < 0.05 was regarded as significant difference between groups.

SPSS 24.0 software (Version 24.0, USA) was used to conduct statistical analysis of data (USSPSS Inc.). First of all, the Kolmogorov-Smirnov method was tested for the normal distribution; then the variance homogeneity test was carried out; and, finally, the one-way ANOVA analysis was carried out. All results were expressed as mean ± standard deviation (M ± SD). In the statistical analysis, *p* value < 0.05 was considered the statistically significant difference.

## 5. Conclusions

Different tonal music enrichment environments induced changes in the expression levels of BDNF and its downstream pathways related to genes and proteins affecting neuronal morphology. The enrichment environment of D-tone music might be conducive to the development of brain nerves in early-life mice.

## Data Availability

Original data used and generated in this study are available from the corresponding authors on request with a completed Data Transfer Agreement.

## Supplementary Materials

The following supporting information can be downloaded at: https://www.mdpi.com/article/10.3390/ijms24098119/s1, Figure S1: BDNF and its downstream effects neuro-plasticity of mice; Table S1: Nutritional composition of breeding feed for mice; Table S2: The analysis of dendrite spines in the hippocampus; Table S3: The analysis of dendrite spines the prefrontal cortex.
