# Supplementary material for: Music with Different Tones Affects the Development of Brain Nerves in Mice in Early Life through BDNF and Its Downstream Pathways
Source: Int J Mol Sci. 2023 May 1;24(9):8119. doi: 10.3390/ijms24098119 (PMC10179650; doi:10.3390/ijms24098119)

Table S1 Nutritional composition of breeding feed for mice

Product	Crude protein	Crude fat	Crude fiber	Crude ash	Water	Calcium	Phosphorus	Calcium: Phosphorus
Breeding feed for mice	⩾200g	⩾40g	⩽50g	⩽80g	⩽100g	10-18	6-12	1.2∶1-1.7∶1

Table S2 The analysis of dendrite spines in the hippocampus

Group	Number of dendritic spines	Length of dendritic spines (um)	Number of dendritic spines per unit length (number/10um)
C	16[a]	43.478[c]	3.68002208[a]
D	25[c]	39.0599[b]	6.400426012[b]
A	21[b]	33.404[a]	6.286672255[b]
G	26[c]	35.195[a]	7.387412985[b]

Note: Different lowercase showed the statistical significance between different groups

Table S3 The analysis of dendrite spines in the prefrontal cortex

Group	Number of dendritic spines	Length of dendritic spines (um)	Number of dendritic spines per unit length (number/10um)
C	12[a]	38.0539[b]	3.153421857[a]
D	24[c]	40.1726[c]	5.974221235[b]
A	21[b]	38.1158[b]	5.509526233[b]
G	22[b]	36.1808[a]	6.080573122[b]

Note: Different lowercase showed the statistical significance between different groups

Figure S1BDNF and its downstream effects neuro-plasticity of mice

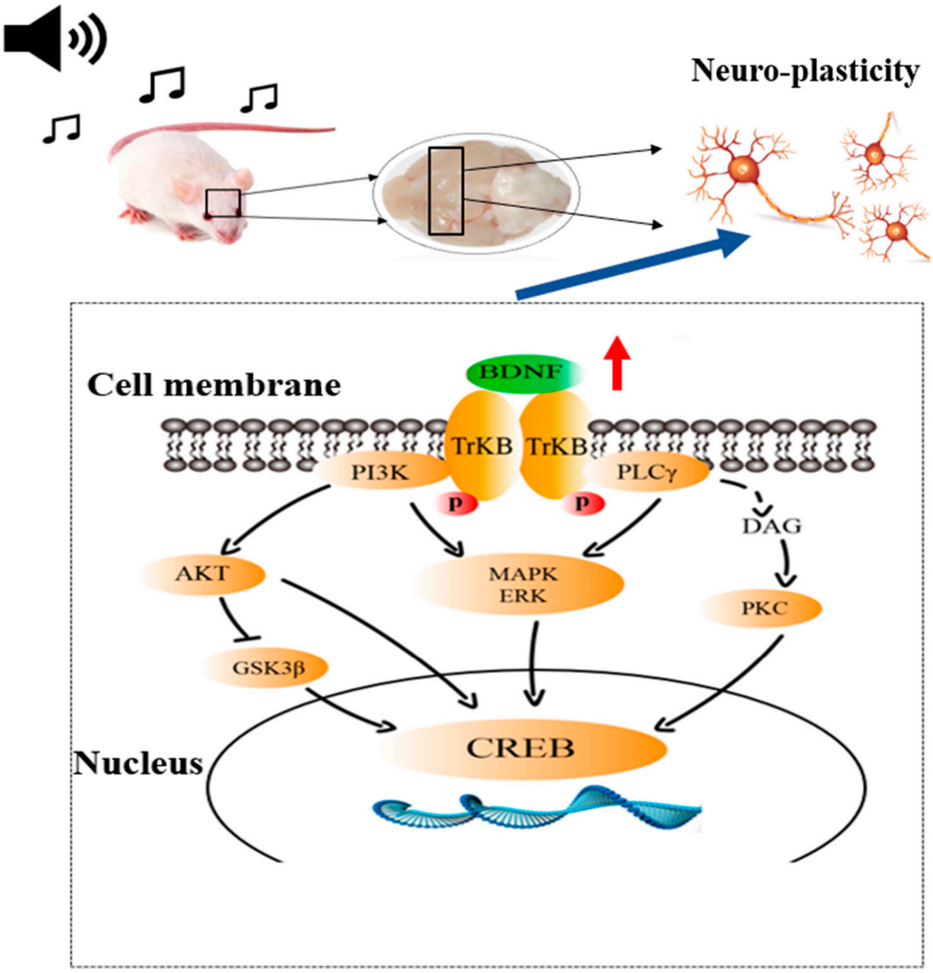

Supplement: Supplementary file 1 [file ijms-24-08119-s001.zip › ijms-2335069-supplementary.pdf]
